# Latin American expert opinion paper on the diagnosis and treatment of pulmonary hypertension associated with interstitial lung disease

**DOI:** 10.3389/fmed.2026.1798347

**Published:** 2026-05-26

**Authors:** Adrián Lescano, Tomás Pulido Zamudio, Rafael Conde Camacho, Fabián Matías Caro, Guadalupe Espitia Hernández, Caio Julio Cesar dos Santos Fernandes, José Antonio García Cuéllar, Mónica Zagolín Blancaire, Nayeli Zayas Hernández, Manuela Tobón

**Affiliations:** 1 Cardiology Department, Fundación Favaloro/Centro Gallego, Buenos Aires, Argentina; 2 National Institute of Cardiology Ignacio Chávez, Ciudad de México, Mexico; 3 Pulmonary Vascular Disease Program, Fundación Neumológica Colombiana, Bogotá, Colombia; 4 Interstitial Lung Disease Section, Respiratory Rehabilitation Hospital "María Ferrer", Buenos Aires, Argentina; 5 Primero de Octubre Regional Hospital, ISSSTE, Ciudad de México, Mexico; 6 INCOR and Sirio Libanés Hospital, São Paulo, Brazil; 7 Pulmonary Hypertension Clinic, National Institute of Respiratory Diseases "Ismael Cosío Villegas" (INER), Ciudad de México, Mexico; 8 Pulmonary Hypertension Programme, National Thoracic Institute and Clínica Santa María, Santiago, Chile; 9 University of Chile, Santiago de Chile, Chile; 10 Cardio VID Clinic, Medellín, Colombia

**Keywords:** algorithm, diagnosis, interstitial lung disease, PH-ILD, pulmonary hypertension, treatment

## Abstract

Pulmonary hypertension associated with interstitial lung disease (PH-ILD) represents a major clinical challenge due to its impact on prognosis and the complexity of diagnosis and management. The objective of this work was to develop practical, region-specific guidelines primarily aimed at supporting healthcare professionals in Latin America. A multidisciplinary working group of Latin American experts in PH-ILD developed an expert opinion paper, from which a diagnostic and therapeutic algorithm was derived. The resulting algorithm provides a stepwise approach to identifying suspected PH in patients with interstitial lung disease, confirming the diagnosis, and guiding management and treatment. Clinical, functional, biological, and imaging findings, including transthoracic Doppler echocardiography, are integrated to estimate the probability of PH, with tricuspid regurgitation velocity used to stratify risk. Right heart catheterization is proposed to confirm the diagnosis and to inform treatment decisions according to current recommendations. This algorithm offers a structured and practical tool that may facilitate earlier diagnosis and support appropriate management of patients with PH-ILD in routine clinical practice.

## Introduction

Interstitial lung disease (ILD) includes a large group of more than 200 parenchymal lung disorders, most of which are classified as rare diseases. Treatment options vary depending on the aetiology, so it is important to properly define its classification (1–5). Similar to idiopathic pulmonary fibrosis (IPF), several other ILDs (such as hypersensitivity pneumonitis, ILD associated with autoimmune diseases, or unclassifiable idiopathic interstitial pneumonia [IIP]) may present with a progressive fibrosing phenotype (1–4). This progressive fibrosing phenotype is presented mainly by the following ILDs: idiopathic non-specific interstitial pneumonia, unclassifiable IIP, interstitial pneumonia with autoimmune features, rheumatoid arthritis ILD, systemic sclerosis ILD, hypersensitivity pneumonitis, sarcoidosis, and ILDs related to other occupational exposures (e.g., asbestosis, silicosis) (1). IPF is the most studied and common ILD. It is characterized by progressive fibrosis, pulmonary scarring, and a radiological pattern known as ‘usual interstitial pneumonia’ (1–4). Progressive fibrosis of the lung parenchyma is self-sustaining, causes progressive worsening of lung function and respiratory symptoms, with poor quality of life and survival rates (1).

Even though there are no uniformly accepted criteria for defining whether an ILD has a progressive phenotype (1), in 2022 the ATS/ERS/JRS/ALAT published international guidelines on the diagnosis and treatment of progressive pulmonary fibrosis, proposing that at least two of the following criteria occurring within the previous year with no alternative explanation must be met: (1) worsening respiratory symptoms, (2) physiological evidence of disease progression, and (3) radiological evidence of disease progression (6–9).

Worsening of respiratory symptoms is defined as worsening of dyspnoea that cannot be explained by any other cause. The physiological evidence of disease progression is defined as the presence of any of the following findings if they are attributable to worsening fibrosis: the absolute decrease in FVC of >5% within 1 year of follow-up or the absolute decrease in DLCO (corrected for Hb) of >10% every 6 to 12 months of follow-up. The radiographic evidence of disease progression may include one or more of the following: (1) increasing extent or severity of traction bronchiectasis and bronchiolectasis, (2) new ground-glass opacity with traction bronchiectasis, (3) new fine reticulation, (4) increasing extent or coarsening of the reticular abnormality, (5) new or increased honeycombing or (6) increasing volume loss (6–9).

Pulmonary hypertension (PH) is a pathophysiological disorder characterized by high pulmonary pressure and vascular remodelling with increased pulmonary vascular resistance (PVR), associated with some cardiac, respiratory, and immunological diseases, among others (10). PH is defined by a mean pulmonary artery pressure (mPAP) higher than 20 mmHg at rest. It is classified into 5 subgroups: Group 1—Pulmonary arterial hypertension; Group 2—PH associated with left heart disease; Group 3—PH due to chronic lung disease and/or hypoxia; Group 4—Chronic thromboembolic PH and other pulmonary artery obstructions; and Group 5—PH of undetermined or multifactorial origin (10). PH is frequently observed in patients with ILD, COPD, and/or emphysema, combined pulmonary fibrosis, emphysema, and hypoventilation syndromes. In ILD, the risk of developing PH varies depending on the aetiology.

PH associated with ILD (PH-ILD) is included in group 3 of the classification of PH, is observed mostly in patients with progressive pulmonary fibrosis, and it is what the present manuscript focuses on. The prevalence of group 3 is approximately 4 per 10,000, and of PH-ILD between 0.8–1 per 10,000 (11). Group 3 PH has the lowest survival of the five groups. In the United Kingdom, a longitudinal cohort of PH showed the shortest median survival (approximately 21 months) for group 3 PH compared with other PH groups (11). The presence of PH regardless of severity has been shown to negatively impact survival in both COPD and ILD; within group 3 PH, PH-ILD is the most severe subgroup and carries the highest mortality. Group 3 PH, and by extension PH-ILD, are therefore serious diseases, and are unique among PH groups due to their substantial clinical burden (11). The recommendations in the 2022 ESC/ERS guidelines and the publications of the 7^th^ ESC World Symposium on Pulmonary Hypertension indicate that PH should be confirmed by right heart catheterization (RHC). RHC is essential for diagnosing and establishing the phenotype of PH-ILD, and accordingly it should be considered to establish prognosis and for treatment decision-making (12). Once PH has been diagnosed, recommended treatment includes supplemental oxygen therapy, and pulmonary rehabilitation (10, 11, 13–15). Previous clinical trials evaluating pulmonary vasodilator therapies in patients with PH-ILD have shown negative or inconclusive results, particularly with endothelin receptor antagonists and other agents (16–18). These findings highlight the complexity of treating PH-ILD and underscore the need for careful patient selection and individualized therapeutic decision-making (10, 17, 19, 20). Supported by the INCREASE study, the use of inhaled treprostinil has demonstrated improvements in these patients and may be considered as treatment (10–12, 17, 19, 20), although more information is needed. Importantly, cases of severe PH should be referred to a PH specialized centres for individualized care (10–12).

There are no specific guidelines that include recommendations for diagnosis and treatment of PH-ILD adapted to the Latin American context. In Latin America, the diagnosis and management of PH-ILD are further challenged by the marked heterogeneity of healthcare systems across countries. Access to specialized centres, RHC, advanced imaging techniques, and lung transplantation programmes varies considerably within the region. In addition, differences in healthcare coverage and reimbursement policies may limit access to specific pharmacological treatments, contributing to variability in clinical practice. These disparities highlight the need for adaptable clinical approaches that can be implemented across diverse healthcare settings (21, 22).

There are many unknowns about the management of patients with PH-ILD, due to its great complexity. Therefore, the objective of this review was to establish guidelines that enable healthcare professionals to make the best therapeutic decisions in the Latin American setting. For this purpose, published scientific evidence was reviewed, together with the knowledge and experience of experts in cardiology and pulmonology in Latin America, who jointly developed this expert opinion paper.

## Methods

A multidisciplinary working group was formed, consisting of specialist clinicians in cardiology and pulmonology experts in PH-ILD from several Latin American countries (Argentina, Brazil, Chile, Colombia, and Mexico). Two internationally renowned pulmonology experts (Dr. Vicent Cottin and Dr. Moises Selman) also collaborated as advisors.

The group of experts participated in three working meetings, from February to June 2025. One meeting was in person and two were virtual. In successive meetings and rounds of review, a shared document was developed, using an online platform that allowed for collaborative work on the development, review and validation of the definitive algorithm for diagnosis and management of ILD-PH in Latin American patients presented in this article.

## Results

**Figure 1:**
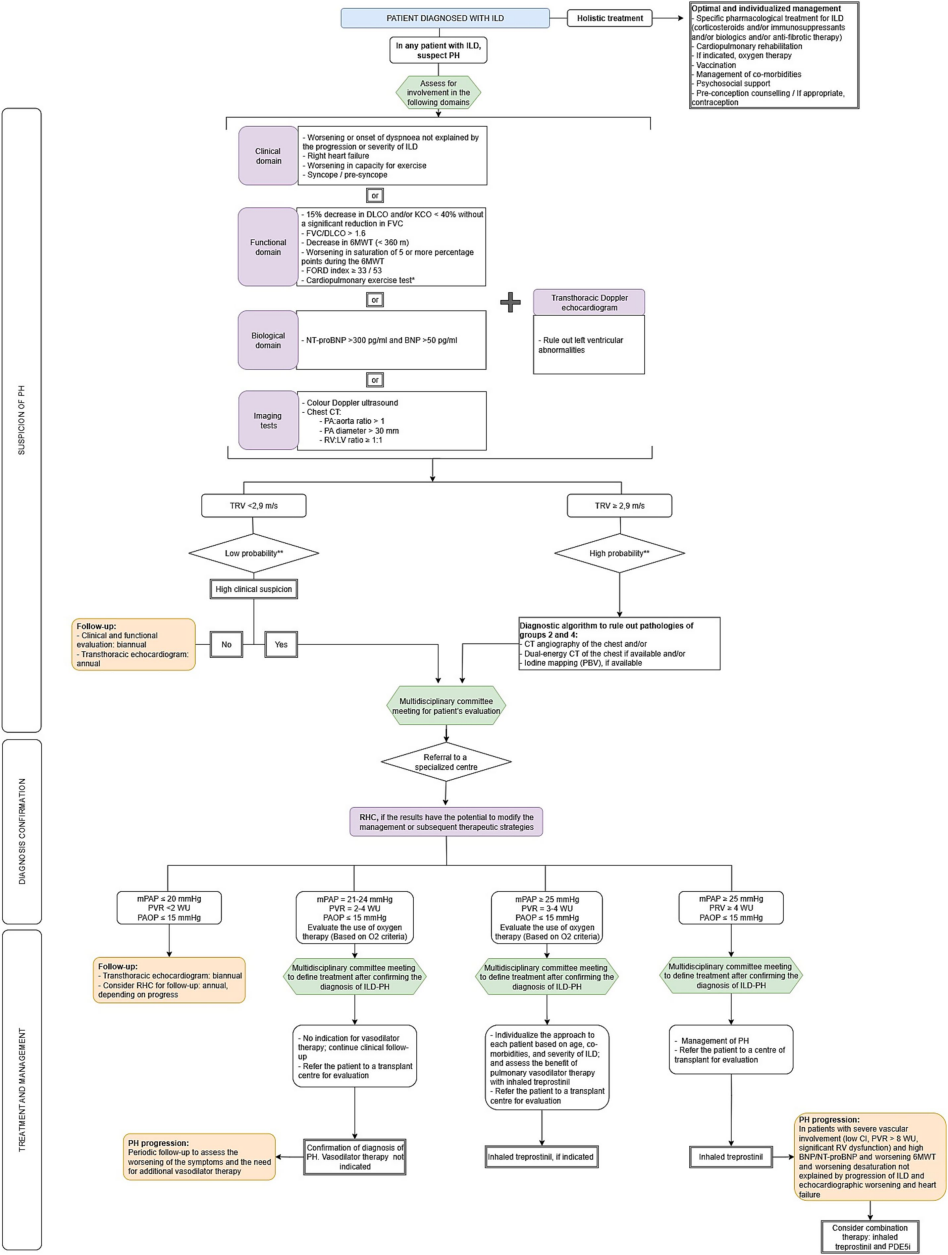
Algorithm for diagnosis and management of PH-ILD in Latin American patients. * In centres where cardiopulmonary exercise test is available, a decrease of VO_2_ peak, combined with evidence of ventilatory inefficiency such as an increased VE/VCO_2_ slope, a flat or falling PETCO_2_, and a positive PaCO_2_-PETCO_2_ difference, suggests the presence of PH. ** The echocardiographic probability of PH is interpreted in the same way as in the other PH groups, based on TRV and right-chamber changes. BNP: B-type natriuretic peptide; CI: cardiac index; CT: computed tomography; DLCO: diffusing capacity for carbon monoxide; FORD: (FVC%/DLCO%, Oxygenation, Race, and Distance); FVC: forced vital capacity; ILD: interstitial lung disease; PH-ILD: pulmonary hypertension associated with interstitial lung disease; KCO: carbon monoxide transfer coefficient; LV: left ventricle; mPAP: mean pulmonary arterial pressure; NT-proBNP: N-terminal pro-B-type natriuretic peptide; O_2_: oxygen; PA: pulmonary artery; PaCO_2_: partial pressure of carbon dioxide; PAOP: pulmonary artery occlusion pressure; PBV: perfused blood volume; PDE5i: phosphodiesterase 5 inhibitor; PETCO_2_: partial pressure of end-tidal carbon dioxide; PH: pulmonary hypertension; PVR: pulmonary vascular resistance; RHC: right heart catheterization; RV: right ventricle; 6MWT: six-minute walk test; TRV: tricuspid regurgitation velocity; VE/VCO_2_: ventilation/carbon dioxide production slope; VO_2_: peak oxygen consumption rate; WU: Wood unit.

The experts agreed that all patients with ILD should be assessed for risk of developing PH, and they proposed the algorithm shown in Figure 1. The review-based points in this algorithm are based on published scientific evidence and the expertise and clinical experience of the panel of experts. Each point of the algorithm is explained and justified in detail in the discussion, together with previously published evidence on the matter.

## Discussion

### Assessment of ILD aetiology

Since the aetiology of ILD is variable, and affects the risk of developing PH as well as its severity, it is necessary to understand the ILD aetiology of each patient, in order to assess the suspicion of PH associated with ILD, and focus the diagnostic process (11). Understanding the aetiology of ILD in these patients will also enable to choose the therapeutic strategy for managing their condition that best suits their characteristics. Treatment should be approached from a holistic perspective, that is, not only pulmonary symptoms should be considered, but also the patient as a whole (4, 23) and the question of what measures are appropriate for each case should be raised: specific pharmacological treatment for ILD (corticosteroids, immunosuppressants, biologics, anti-fibrotic therapy), cardiopulmonary rehabilitation, oxygen therapy, vaccination, management of co-morbidities (diabetes, heart failure, sleep disorders, etc), psychosocial support, nutritional advice, and pre-conception /contraception advice, if applicable.

It is essential that physicians who are not specialists in PH-ILD, but care with patients with ILD, possess a general understanding of the underlying disease, particularly of the more common forms, that are likely to be associated with complications such as PH. This ensures that, by the time the patient reaches the vascular disease specialist, there is already a clinical path with a well-established and substantiated diagnosis. Not all cases of ILD carry the same risk of developing PH: while anti-synthetase syndrome is rarely associated with PH, disorders such as systemic sclerosis, rheumatoid arthritis, sarcoidosis or mixed connective tissue disease have a high prevalence of PH. In IPF, the frequency of PH is higher than in other ILDs: it has been described in 8–15% of these patients at the initial examination (10). In autoimmune diseases, the association between ILD and PH is primarily related to pulmonary and/or vascular involvement (1, 10, 24) and in these diseases, the origin are especially relevant, and they require a coordinated diagnostic and therapeutic approach from an early stage (1). PH is also a common complication of combined pulmonary fibrosis and emphysema (CPFE) and sarcoidosis. Patients with these diseases have an increased PH risk (prevalence of 28% and, prevalence up to 50% and severe PH 70% respectively) (25) compared with those with IPF (OR: 2.66; 95% CI: 1.55–4.57; *p* < 0.01) or emphysema (OR: 3.19; 95% CI: 1.42–7.14; *p* < 0.01), respectively (26).

### Suspicion of PH

It is crucial to understand that PH can appear at any time during the course of ILD. Although its prevalence tends to be higher in patients with more extensive and severe interstitial involvement, it should not be assumed that this complication only appears in advanced stages (10, 23). Consequently, all patients with ILD should be evaluated for possible PH or other cardiovascular disease. Physicians must maintain a high level of suspicion to advance the diagnostic process, and not base the diagnosis solely on the severity of the ILD (11, 23). Although not all countries and hospitals have the same resources, there is a series of essential tests to reach the conclusion of suspected PH. Each centre must adapt the algorithm based on its available resources and its situation to detect PH early. There are five domains that should be assessed in these patients, following the algorithm developed in this review: clinical situation, pulmonary function exams, biological parameters, imaging tests, and transthoracic echocardiogram. The choice of diagnosis tests and which domains are chosen will depend on the access and resources of each centre.

Clinical worsening of ILD is a criterion for suspicion of PH (9, 23, 27): This includes new or worsening dyspnoea that cannot be explained by the progression or severity of ILD, signs of right heart failure, reduced exercise capacity, and episodes of syncope/pre-syncope. At the same time, deterioration in lung function is a key criterion for screening for PH (9, 27): decrease in DLCO ≥ 15% every 6 to 12 months of follow-up, or DLCO or KCO (carbon monoxide transfer coefficient) < 40% in the absence of a parallel decrease of FVC; FVC/DLCO > 1.6; reduced 6MWT (six-minute walk test) < 360 m; worsening in oxygen saturation of 5% or more during the 6MWT every 6 to 12 months of follow-up; and value of FORD (FVC%/DLCO%, Oxygenation, Race, and Distance) index ≥ 33, which provides a good balance of sensitivity and specificity for predicting PH, and FORD index ≥53, which is associated with a very high specificity (98%) for PH (28). Other complementary tests may contribute to the diagnosis of PH, if available (9, 23, 27). In centres where cardiopulmonary exercise testing (CPET) can be performed, it may be used as an optional complementary tool to support the suspicion of PH, to assess whether patients’ dyspnoea and exercise limitation are due to ventilatory, cardiovascular or pulmonary vascular causes; however, it is important to note that this tool is not available in all centers (29–31). Parameters such as a decreased VO_2_ (oxygen consumption rate) peak or decreased oxygen pulse, combined with data indicative of ventilatory insufficiency such as an increase in the VE/VCO_2_ (ventilation/VCO_2_) slope, a flat or downward pattern of PETCO_2_ (partial pressure of end-tidal carbon dioxide), or a positive PaCO_2_-PETCO_2_ (partial pressure of carbon dioxide **-** PETCO_2_) difference, suggest the presence of PH (32). In this proposed algorithm, two biological parameters were also agreed upon: the high levels of NT-proBNP and BNP. According to the established criteria for group 1 of PH, NT-proBNP levels > 300 pg./mL and BNP > 50 pg./mL increase suspicion of PH, and this could be extrapolated to PH-ILD (9, 27). Regarding imaging tests, colour Doppler ultrasound and chest CT were agreed upon. The CT scan identifies signs suggestive of PH, such as pulmonary artery dilation, while the transthoracic echocardiogram assesses cardiac repercussions (9, 23, 27): PA/aorta ratio > 1, PA diameter > 30 mm, and enlarged right cardiac chambers.

The experts agreed that all patients with ILD should undergo a transthoracic echocardiogram. Heart failure with preserved ejection fraction coexisting in patients with group 3 PH — estimated at between 10 and 30%—frequently acts as a comorbidity rather than a dominant factor in the pathogenesis of PH (33). So it is essential to rule out left ventricular abnormalities, especially diastolic dysfunction with normal ejection fraction, by using echocardiographic parameters that estimate filling pressures of the left ventricle, and guide diagnosis and appropriate management (34). Depending on the availability of resources in each country, transthoracic echocardiogram may be the first step in the evaluation of patients with ILD, or it could be considered as an additional domain to the other tests to assess suspicion of PH, independent of the results of the other domains. Furthermore, in places with a greater accessibility, an echocardiogram may be considered as part of the routine follow-up in patients with ILD, given that they have a higher burden of cardiovascular co-morbidities (35). Either way, an echocardiogram should be a basic requirement, not an option, as it is essential to assess tricuspid regurgitation velocity (TRV) and tricuspid S′ wave velocity, right heart chambers and dilation of the left atrium, systolic and diastolic function, valvular heart disease, and cardiac structural abnormalities from the outset. Although some centres lack the resources to perform echocardiograms, their universal implementation should be prioritized, thus avoiding suboptimal alternatives that delay diagnosis (23, 27). In addition, a transthoracic echocardiogram provides information about TRV and indirect parameters about right ventricle (RV) dysfunction, including tricuspid annular plane systolic excursion (TAPSE) < 16, right ventricle fractional area change, inferior vena cava collapsibility, right ventricle/left ventricle ratio, Myocardial Performance Index (MPI), E/A ratio as well as dilation of the right atrium and right ventricle (36). In PH-ILD, a TAPSE/sPAP ratio < 0.55 mm/mmHg is considered an additional echocardiographic sign of PH, according to the 2022 guidelines. Furthermore, a cut-off of < 0.32 mm/mmHg is used for intermediate-high risk of mortality and < 0.19 mm/mmHg for high risk of mortality (10).

Based on the results obtained from the echocardiogram, together with the assessment of the other domains, patients can be classified according to their probability of suffering from PH (10, 23): if the TRV is less than 2.9 m/s, probability is low; and if TRV is ≥ 2.9 m/s, probability is high. Patients with a low probability but a high clinical suspicion of PH-ILD (defined as proactive vigilance: systematically looking for the disproportionate decline in DLCO, exercise capacity, or new right-heart signs in an ILD patient, then escalating to echocardiography promptly rather than attributing everything to parenchymal progression) (37) should be evaluated in the same way as patients with a high probability. However, those without a high clinical suspicion will be followed every 6 months, to detect possible changes in clinical symptoms and lung function, and a transthoracic echocardiogram will be repeated in these patients as an annual follow-up. Patients with a high probability of developing PH should be evaluated to rule out group 2 and 4 pathologies, using chest CT angiography and/or Dual-energy chest CT (10). All patients should be evaluated in multidisciplinary meetings ideally composed of pulmonologists, cardiologists, rheumatologists, radiologists, a physical and pulmonary therapist, a nutritionist, a psychologist and a coordinating nurse, to make individualized and appropriate decisions for each case. All patients with high probability of developing PH, or patients with a low probability but who fulfil the clinical suspicion criteria discussed above, will be considered as patients with suspicion of PH. They will be referred to specialized centres to proceed with RHC to continue the process of confirming the diagnosis (11).

### Confirmation of PH

RHC remains crucial for confirming the diagnosis of PH-ILD (10). RHC is recommended in patients with suspicion of PH in whom the catheterization result could modify their management or the subsequent therapeutic strategies. It should be avoided in clinical conditions in which catheterization is contraindicated, such as those in patients in functional class IV on high-flow oxygen therapy and with limited life expectancy, patients with current infections, right heart failure or exacerbations, because the risk–benefit ratio does not justify the procedure. In extreme clinical settings, such as acute right heart failure or severe respiratory exacerbations, the RHC should be postponed, because the procedure could aggravate haemodynamic instability. In these cases, the patient’s stabilization should be prioritized before performing the RHC study (27). Instead, it should be prioritized in patients with intermediate/high haemodynamic risk identified by echocardiography, especially when there is a discrepancy between symptoms and non-invasive findings. In centres without technical experience, referral to specialized units is highly recommended to minimize risks and guarantee accurate measurements, ensuring that the indication always aligns with the expected clinical benefit (38).

The diagnosis of PH-ILD will be confirmed when the catheterization result is mPAP ≥ 20 mmHg and PVR ≥ 2 WU (Wood unit), and pulmonary arterial occlusion pressure (PAOP) ≤ 15 mmHg in pre-capillary PH-ILD (10). If these criteria are not fulfilled, it is recommended to repeat the echocardiogram every 6 months and perform annual follow-up of the established diagnostic criteria for clinical symptoms and lung function (32, 39–41). Annual RHC may be considered, depending on the evolution of the symptoms and the screening signs.

### PH-ILD treatment

To decide upon the most appropriate treatment strategy in PH-ILD, it is essential that the multidisciplinary team at the specialized centre meet and jointly evaluate the case. Treatment should be approached from a holistic perspective, that is, management of the ILD as mentioned above and management of PH (23, 42). In this context, general supportive measures are essential components of care in patients with PH-ILD. These include pulmonary rehabilitation, oxygen therapy when indicated, smoking cessation, vaccination, and comprehensive management of comorbidities (such as cardiovascular disease, sleep disorders, and metabolic conditions), as well as psychosocial support and nutritional counselling. These measures play a key role in improving functional capacity, quality of life, and overall clinical outcomes, and should be systematically considered in all patients (40).

In addition, all patients with a confirmed diagnosis of PH-ILD should be referred to a transplant centre to assess their risk, and to determine whether they are candidates for a lung transplant. Likewise, in all these cases, supplementary oxygen therapy should be considered in each case, based on the established criteria for chronic hypoxemia (PaO_2_ ≤ 55 mmHg, or SpO_2_ ≤ 88% at rest, or PaO_2_ 56–59 mmHg with signs of PH, or secondary polycythaemia), to confirm whether the patients meet the characteristics to receive oxygen therapy (43).

The management of PH in PH-ILD patients will depend on the results of the RHC (10, 42). If mPAP = 21–24 mmHg, PVR = 2–4 WU, and PAOP ≤ 15 mmHg, the diagnosis is confirmed, but vasodilator drug is not indicated. In these cases, follow-up will be performed to assess the worsening of PH symptoms, in order to reconsider PH specific anti-remodelling therapy if the conditions for its indication are met in the future (44). In patients with mPAP ≥ 25 mmHg, PVR = 3–4 WU, and PAOP ≤ 15 mmHg, the diagnosis is confirmed, and it is recommended to individualize the approach to each patient based on age, co-morbidities, aetiology and severity of ILD, FVC, and/or other factors, assessing the potential benefit of treatment with inhaled treprostinil (44). In patients with mPAP ≥ 25 mmHg, PVR ≥ 4 WU, and PAOP ≤ 15 mmHg, treatment with inhaled treprostinil may be considered (Table 1). Evidence has shown an improvement of 6WT 31 meters, 15% decrease in NT-proBNP and a 40% risk reduction of clinical worsening. The *post hoc* subgroup analyses in INCREASE study showed that the treatment with inhaled treprostinil at a dose of >9 bps resulted in fewer clinical worsening events and greater clinical improvement (44–46).

**Table 1:** Recommended therapeutic actions based on RHC results and levels of evidence of the recommendations.

Hemodynamic profile	Confirmation of diagnosis	Recommended action	Level of evidence
mPAP ≤ 20 mmHg PVR = < 2 WU PAOP ≤ 15 mmHg	Diagnosis of PH not confirmed	Follow-up: biannual transthoracic echocardiogram and consider annual RHC	2022 ESC/ERS guidelines (10) and expert opinion
mPAP = 21–24 mmHg PVR = 2–4 WU PAOP ≤ 15 mmHg	Confirmation of the diagnosis of PH	Vasodilator drug is not indicated. Follow-up: assessing the worsening of PH symptoms, to reconsider PH specific vasodilator therapy in the future	2022 ESC/ERS guidelines (10) and expert opinion
mPAP ≥ 25 mmHg PVR = 3–4 WU PAOP ≤ 15 mmHg	Confirmation of the diagnosis of PH	Individualized approach to each patient based on age, co-morbidities, aetiology and severity of ILD, FVC, and/or other factors. Assess the potential benefit of treatment with inhaled treprostinil	Clinical trial evidence (INCREASE) (44–46) and expert opinion
mPAP ≥ 25 mmHg PVR ≥ 4 WU PAOP ≤ 15 mmHg	Confirmation of the diagnosis of PH	Treatment with inhaled treprostinil may be considered	Clinical trial evidence (INCREASE) (44–46) and expert opinion
mPAP ≥ 25 mmHg PVR > 8 WU PAOP ≤ 15 mmHg	Confirmation of the diagnosis of PH	Combination treatment with inhaled treprostinil and PDE5i may be considered	Clinical trial evidence (INCREASE) (44–46), 2022 ESC/ERS guidelines (10) and expert opinion

mPAP, mean pulmonary arterial pressure; PAOP, pulmonary artery occlusion pressure; PDE5i, phosphodiesterase 5 inhibitor; PH, pulmonary hypertension; PVR, pulmonary vascular resistance; RHC, right heart catheterization; WU, Wood unit.

In countries in which this therapy is not available, although the use of a phosphodiesterase 5 inhibitor (PDE5i), such as sildenafil, might be considered as an alternative, there is limited evidence of its effectiveness and a recommendation for or against cannot be made. In the event of worsening of the patient’s condition with high BNP/NT-proBNT (BNP: B-type natriuretic peptide/N-terminal pro-B-type natriuretic peptide) and/or worsening in 6MWT, desaturation not explained by progression of ILD, echocardiographic abnormalities, and/or heart failure, combined treatment with inhaled treprostinil and a PDE5i might be considered (10). According to the evidence, there are no direct data from clinical trials about the safety, tolerability, and efficacy of PDE5i (such as sildenafil) in patients with PH-ILD. Indirect data included in the guidelines do not allow to extract firm conclusions. Given the lack of solid evidence, the ERS/ESC Working Group, in the 2022 guidelines (10), was unable to formulate a recommendation for or against the use of PDE5i in patients with PH-ILD and recommended that these patients be referred to a PH centre for individualized decision-making. The use of ambrisetan and riociguat is contraindicated in these patients (10). In patients with very high PVR (such as, mPAP ≥ 25 mmHg, PVR ≥ 8 WU, and PAOP ≤ 15 mmHg), and limited extent of their ILD that does not account for such haemodynamic change at the pulmonary level, according to their individual characteristics, their medical history, comorbidities, and extent of ILD, the possibility that their PH is of group 1 aetiology should be considered. In this case, treatment should be in accordance with the evidence available from group 1, bearing in mind that both riociguat and ambrisentan are contraindicated in these patients. Such cases should be evaluated individually and by multidisciplinary teams in specialized centres (10) (Table 1).

The marked heterogeneity of healthcare systems and treatment access across Latin American countries significantly impacts management, and prognosis of patients with PH-ILD. For instance, the availability of inhaled treprostinil varies between Latin American countries, which represents a relevant distinction and reinforces the clinical applicability of the proposed algorithm in this setting (21, 22).

In this context, the expert panel considered it essential to define an ideal and comprehensive clinical pathway that reflects best practice when all resources are available while allowing flexible and pragmatic implementation according to the specific characteristics of each healthcare system.

This document presents the limitations and biases inherent in expert opinion papers. Furthermore, there was a geographical limitation among the participating experts, as they came from only five Latin American countries. However, the review also presents some strengths: the expertise of participants on the subject, including Dr. Cottin and Dr. Selman, two internationally renowned pulmonology experts as advisors, and the fact that the circumstances that may arise in Latin America were taken into account in the development of this expert opinion paper.

In conclusion, we believe that the presented algorithm can facilitate the diagnosis, management and treatment of patients with PH-ILD.

## Data Availability

The original contributions presented in the study are included in the article/supplementary material, further inquiries can be directed to the corresponding authors.
